# A phenomenological study on barriers of adherence to medical advice among type 2 diabetic patients

**DOI:** 10.1186/s12902-021-00928-x

**Published:** 2022-01-06

**Authors:** Monire Davoodi, Behnaz Dindamal, Hossein Dargahi, Farzad Faraji-Khiavi

**Affiliations:** 1 Department of Health Services Management, School of Health, Ahvaz Jundishapur University of Medical Sciences, Ahvaz, Iran; 2 Health Information Management Research Center, Tehran University of Medical Sciences, Tehran, Iran; 3 Social Determinants of Health Research Center, Ahvaz Jundishapur University of Medical Sciences, Ahvaz, Iran

**Keywords:** Medical advice, Type 2 diabetes, Community Health Center

## Abstract

**Background:**

More than three decades of research and study for overcoming the problem of “non-acceptance/non-compliance” of patients has neither resolved nor reduced the severity of this problem. This phenomenological study aimed to identify barriers of adherence to medical advice among type 2 diabetic patients.

**Methods:**

This study was a qualitative research using phenomenology approach, and the data were analyzed using content analysis approach. Participants were 69 type 2 diabetic patients covered by the diabetes unit of West and East Community Health Centers of Ahvaz, Iran. The views and attitudes of patients about the barriers of adherence to medical advice were elicited by conducting 20–45 min sessions of semi-structured interviews. Data analysis was performed following Colaizzi’s seven-step method.

**Results:**

Barriers of adherence to medical advice were classified into systemic and individual barriers. Individual barriers included 11 codes and 5 categories, and systemic barriers contained within 5 codes and 3 categories. Physiologic and physical factors, financial problems, occupational factors, attitudinal problems and lack of knowledge, and social and family problems were identified as individual barriers. Systemic barriers included inadequate publicizing and limited notification, inadequate equipment and facilities, and poor inter-sectional coordination.

**Conclusions:**

Generally, problems stated by diabetic patients at the individual level can partly be solved by training patients and the people around them. However, as for the systemic problems, it seems that solving the barriers of adherence to medical advice requires coordination with other organizations as well as intersection coordination. Overall, these problems require not only comprehensive health service efforts, but also the support of policymakers to resolve barriers at infrastructure level.

## Background

Diabetes has affected millions of people around the world, and there are many concerns about the complications of this disease [1]. Since 1980, the number of people living with diabetes has almost quadrupled reaching 422 million adults, most of whom living in developing countries [2]. Within the last decade, the number of people living with diabetes has increased at a faster rate in low and middle-income countries compared with higher income countries [3]. Among diabetics, 90–95% have type 2 diabetes [1]. In Iran, the prevalence of diabetes in adults aged 25–70 years was reported to be 11.9% (2011) which shows a 35% increase compared to 2005 [4].

Effective management and control can significantly reduce the risk of disease complications. However, numerous studies conducted across the world have revealed inappropriate control programs and lack of management with regard to diabetes [5–8]. Type 2 diabetes is a chronic disease that affects all aspects of a person’s life. Therefore, the major part of the responsibility for controlling and managing the disease rests on the individual suffering from the disease. In the meantime, findings of several studies point to the fact that 30–50% of patients have poor adherence/acceptance to therapeutic advice [9–11]. More than three decades of research and study for overcoming the problem of “non-acceptance/non-compliance” of patients has neither resolved nor reduced the severity of this problem [9]. For instance, the level of insufficient glucose control in Brazil is 76%. The same figures for Germany, Denmark and Kenya are 40%, 51% and 61%, respectively. These high rates indicate lack of patient adherence to medical advice [12].

While most of the burden of controlling the disease rests on the patient’s shoulders, given the importance of controlling and treating the diabetes, the deputy of health has practically started the prevention, control, and screening programs as well as the diagnosis of the disease since the beginning of 2011 in cities with a population of over one million nationwide [13].

According to the staff of the diabetes unit, after implementation of the diabetes prevention and control programs in diabetes units in Ahvaz, one of diabetic patients’ problems has been lack of disease control despite the provision of services and the necessary therapeutic advice from the staff in the diabetes units. This lack of adherence can have many reasons. Given the high prevalence of diabetes in Ahvaz, this research can help diabetic patients solve their problems in adherence to medical advice based on their own statements. Since no study has been conducted on this topic in Khuzestan province, this study was aimed to identify barriers of adherence to medical advice among type 2 diabetic patients using a phenomenological approach.

## Methods

### Study design

This study was a qualitative research using phenomenology approach, and the data were analyzed using content analysis approach. The choice of a phenomenology approach was justified by the fact that, based on a logical assumption, individuals are the best reference for describing situations, feelings, and experiences by using their own words.

### Participants and sampling

The research population was 69 type 2 diabetic patients covered by the diabetes unit of Community Health Centers in Ahvaz. Ahvaz is the capital of Khuzestan province in southwestern Iran, with a population of more than 1.3 million and hot and dry climate. Ahvaz Jundishapur University of Medical Sciences is the main provider of health services in this city. Participants were selected using purposeful sampling. The inclusion criteria were failure in continuous control of blood sugar, lack of regular visits to the clinic, and patient self-declaration of non-adherence to medical advice. Medical records were used to identify these people, and those patients whose disease was not controlled (i.e., their condition deteriorated based on the information in the patient’s medical records) and who did not visit the center regularly, were invited to be interviewed. Patients who did not consent to participate in research or those who had moved to other cities were excluded. In this study, none of the participants wanted to discontinue participation, and all selected patients participated in the interviews.

### Interview and data collection

The data collection method was semi-structured interview. Two researchers (F.F and M.D‌) with good experience in this field were selected as interviewers. The interviews were conducted face to face. The subjects who met the inclusion criteria were called to set an appointment for interview and a visit with a specialist; in addition, a visit with nutrition expert visit was arranged for them in health center on the same day.

 We introduced ourselves and reminded ethical issues for informed consent. The interviews started with more general questions: Are you satisfied with your clinical results? Did you act on the given medical advice? Why didn’t you follow the medical advice and given instructions? and What prevented you from following these pieces of advice? We let the participants freely speak out their answers. We used active listening as well as verbal and nonverbal probes to encourage informants to speak their mind out. After the first few interviews, following the main questions, we probed participants’ thoughts about some delicate points mentioned by other informants. Each interview had a closing question about missing questions and points.

In order to collect the required data, each interview lasted from 25 to 45 min. Data collection continued until data saturation where no new concept could be extracted from interviews. This procedure took 8 weeks (from at the beginning of November 2017 by the end of December 2017). All interviews were recorded using a tape recorder, and were then carefully listened to and transcribed verbatim. An attempt was made to conduct the interviews without any bias and to write only the whole content. The content of the interviews after transcription was provided to patients to confirm their accuracy.

### Data analysis

The data were analyzed following Colaizzi’s seven‑step method. The research team read the transcription of the interviews several times, and after being familiar with the data, they identified significant statements. Then, they extracted and formulated relevant meanings into categories and themes that were common about barriers of adherence to medical advices among participants. In the meantime, the team developed an exhaustive description before producing a fundamental structure of this phenomenon. At the end, this structure was verified by the participants and experts in health center [14]. In addition, further discussions with the qualitative research experts enabled the researchers to be reflexive of assumptions and biases that may have influenced the research process. Data coding was done at two different occasions by two different coders, and then the codes were compared and discrepancies were resolved.

### Ethical issues

After receiving approval from the Research Ethics Committee of Ahvaz Jundishapur University of Medical Sciences (Ref. ID: IR.AJUMS.REC.1396.626), the researchers introduced themselves to the study settings (Diabetes unit of Community Health Centers in Ahvaz, Iran). In addition, before the beginning of the interview sessions, informed consent was obtained from the participants. The participants were clearly briefed that they had the right to withdraw from the study at any time even after signing the informed consent. The participants were informed about the aims of the study and confidentiality of their personal information. In order to keep the interviewees anonymous and to distinguish them from one another in presenting the findings of the study, a code was assigned to each participant.

### Scientific trustworthiness of the results

Lincoln and Guba’s four-criteria (credibility, dependability, confirmability, and transferability) were used to check the trustworthiness of the data [15]. To evaluate and enhance the credibility of the findings, sampling continued until data saturation. Transferability of data was ensured by offering a comprehensive description of the subject, participants, data collection, and data analysis. Also, to increase the dependability of the research results, we used external checking. The confirmability of findings was enhanced via investigator triangulation [16].

## Results

The interviewees included 37 females and 32 males whose age range was 34–73. The level of education of these people ranged from illiterate to bachelor’s degree. The patients had been diagnosed with T2DM (type two diabetes mellitus) for a duration of one to 30 years. Sixteen codes in 8 categories were extracted from the interviews. These categories were classified into 2 themes of individual and systematic barriers of adherence to medical advice. Individual barriers included 11 codes and 5 categories, and systematic barriers involved 5 codes and 3 categories. T2DM patients’ statements about individual barriers of adherence to medical advice in addition to categories and codes are presented in Table 1:

**Table 1:** Individual barriers of adherence to medical advices among diabetic patients

Theme	Categories	Codes	Patients’ remarks
Individual barriers	Physiologic and physical factors	comorbidities	• I suffer from digestive problems so that I cannot eat stuff like legumes and vegetables. • I have anemia which prevents avoidance from some foods such as meat, rice and etc. • I suffer from respiratory disease which prevents me from exercising and walking. In case of long walking, my breath will be stopped. • I suffer from heart disease. I have problem adjusting my diet. • I have anemia and many foods which are recommended to me are not enough for my body’s needs.
		Physical weakness	• I cannot comply with the diet because I have physical weakness and I always feel hungry; however, as far as I can, I act on advice.
		Aging	• I can’t do physical exercise recommendations because I am old. My legs hurt and I can’t walk.
		Problems in chewing fiber foods	• I am not able to chew fiber foods such as fruit and vegetables. I refuse to eat them.
	Financial problems	Economic pressure	• Economic factors and financial problems make me feel stressed, and this leads to worsening diabetes and lack of control on my disease, and I can’t act on medical instructions.
		Large number of family members	• The number of people in my family is large and I have to provide the cost of educating and clothing, which in turn leads to lack of attention to medical advice. • I have a lot of mouths to feed and I don’t have enough time for myself to act on medical advices.
		Low income people	• My husband is retired. I do not have the ability to prepare the recommended foods • I am retired. I do not have the financial power to buy the prescribed medications.
	Occupational factors	Type of Job	• I am a taxi driver. Because of my job, I am always behind the car and I can’t have enough exercise and mobility. • I am a government employee. I have been working for a long time, which leads to lack of mobility in my workplace. Also, I feel weak after long hours without calorie intake.
	Attitudinal problems and lack of knowledge	Wrong believes	• I do not act on medical advice because I know that diabetes is not a threat to my life and it does not cause me any problem.
		Self-treatment	• I use medicinal herbs to reduce my glucose and I do not need to use medications prescribed by a doctor.
	Social and family problems	Problems in adjusting with the diet and different tastes of people	• I can’t fit my diet with other family members. I have to consider the tastes of other family members in cooking. • My wife does not cooperate with me in preparing foods that help control my diabetes. • My family observes the given diet in the early stages of my disease to control my blood glucose, but over time, there was a problem in the taste and coordination of foods. • When I go to a party, I have to eat foods, although I know they cause problems for my health.

A majority of patients pointed to their comorbidities. In addition to physiological and physical diseases, a number of the participants referred to psychological disorders which resulted from diabetes. “I have a lot of stress which leads to lack of control over my disease, despite adherence to medical advice; so, adherence to medical advice doesn’t work for me” (Participant 6). Physical weakness, aging, digestive system problems, and even chewing problem were barriers to following medical advice. “They ask me to do exercise; I can’t walk or move too much. I love to, but I can’t” (Participant 1). Some of the participants stated that they are intolerant of special kinds of food: “I don’t tolerate dairy, it makes me sick” (Participant 10). Some did not tolerate medication: “…that tablets make me sick; I feel sick all the day long” (Participant 3). However, these are just one side of problem. Another side of personal problems is that patients could not afford to adhere to medical advice. Patients’ remarks show that it is not easy at all to act on medical advice while having financial problems. Repeatedly we received answers like “I’m a retiree and I can’t afford prescribed medications and diets” (Participant 8) or “My husband is a retiree, and he is under medication too; we can’t afford for all these medications” (Participant 12). Some of the participants were worried about the escalating costs “Medication expenses are too high and still rising, I don’t know what we’re going to do?“ (Participant 15).

In addition, some wrong thoughts and beliefs get people cold feet to act on medical advice. “These pills are chemical. They aren’t good for health; I think traditional [herbal] medication is better” (Participant 3). “These pills are just plaster, they don’t work” (Participant 27). “I believe that adherence to medical advice is useless. I feel that both adherence and non-adherence to medical advice are the same” (Participant 7). Lack of family and social support was another category extracted from interviews. “My wife can’t make a special food just for me! Children don’t like these diets” (Participant 5). Another participant stated: “The government should provide our medications, why should we pay for medication? We have worked for the government, and now we are retired” (Participant 4).

Table 2 shows the barriers in the health system that prevents T2DM patients from adherence to medical advice.

**Table 2:** Systemic barriers of adherence to medical advice among diabetic patients

Theme	Categories	Codes	Patients’ remarks
Systemic problems	Inadequate publicizing and limited notification	Inadequate publicizing	• It is better to have notification in the mass media. I’m very dissatisfied with the level of notification. I think it is due to poor notifying that people are not sufficiently aware about diabetes. • The diabetes unit does not pay much attention to holding festivals and sessions on diabetes. If these festivals are run, we will be encouraged to act on medical advice.
		Lack of correct and timely notification to the community	• I was unaware of the disease and its dangers. I think there should be more efforts to inform the community. I was unaware of my diabetes till the stage of its progression. • I didn’t know much about the disease until I was diagnosed and referred to the diabetes unit.
	Inadequate equipment and facilities	Insufficient personal monitoring devices	• I do not have a blood glucose monitor, so I couldn’t measure my blood glucose levels or control my disease by medication. • The diabetes unit did not cooperate with me to prepare the blood sugar device.
		Limited access to related facilities	• I do not have access to sports facilities for managing my disease, which leads to non-adherence to medical advice. • It takes one day for me to come to the health center and to have my blood glucose checked after a lot of waiting.
	Poor inter-sectional coordination	Limited insurance coverage	• Foreign medications are not covered by insurance. I can’t afford to buy the prescribed medicines. If the government lets some medications be covered by insurance, the problems of providing medications will be solved and I’ll be able to control my disease. • In my opinion, the government should provide patients more services, including releasing some foreign medications from customs warehousing, which can help diabetic patients to have access to their medicines.

Patients were expecting health sector to inform them about diabetes and help them to recognize it at very early stages. While diabetes screening is routinely done in health centers, they were asking for more extensive programs like National Diabetes Week’s festivals that are running in the parks and public places. Some said that they need blood glucose monitors, and there were others who said: “I have the blood glucose monitor but I can’t afford for its kits. I would monitor my blood glucose, if I had these kits “(Participant 14). Quite a number of participants asked for subsidizing foreign medicines or covering them by public insurance companies: “I was using foreign medicines and it was great. Now they [medicines] are scarce and expensive, so I can’t afford to buy them. And medications made in local pharmaceutical manufacturers don’t agree with my stomach” (Participant 19). Another patient said: “I can’t take Iranian medications and I can’t afford foreign ones. The government should force insurance [companies] to cover them” (Participant 15).

**Fig. 1:**
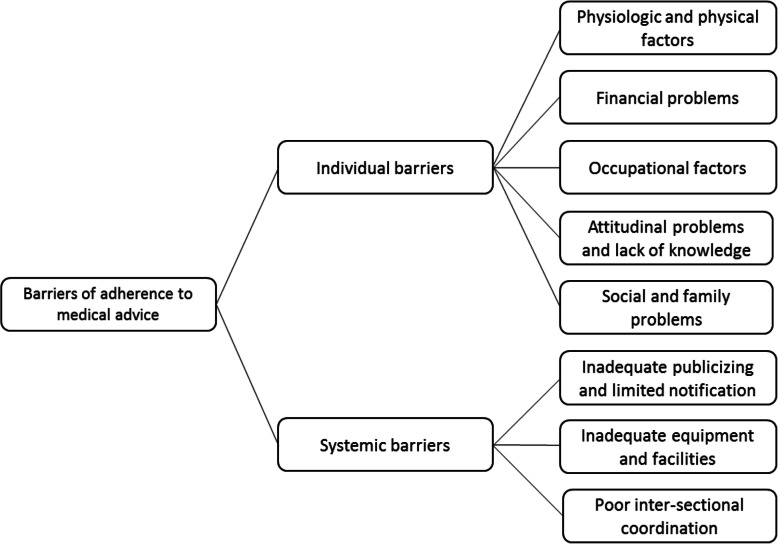
Barriers of adherence to medical advice in diabetic patients in Ahvaz.

In general, the results obtained from the interviews can be summarized in two categories: individual barriers and systemic barriers as depicted in Fig. 1.

## Discussion

The main reasons for T2DM patients’ non-adherence to medical advice can be divided into two major categories. In the first place, there are individual factors that include physical problems, financial problems, occupational factors, attitudinal problems and lack of knowledge, and finally, social and family problems. The second major reason is related to the health system problems which include cases such as limited and inadequate notification, lack of adequate governmental facilities for patients, and lack of inter-sectional coordination. These issues can help the managers in making decisions on how to provide services and remove the existing barriers to increase patients’ adherence to medical advice.

In the conducted interviews, an individual barrier that was repeatedly raised by patients was the physical barriers. According to Worlming et al.’s results, co-morbidity in diabetic patients with diseases such as arthritis, stroke, cardiovascular disease, respiratory diseases, and myocardial infarction was associated with a reduction in physical health, and an increase in the number of comorbid diseases led to a decline in the quality of life, indicating the more pronounced role of doctors in the treatment and health behaviors of diabetic patients [17].

Considering that many patients mentioned stress as an important factor in their failure to follow medical advice, mindfulness-based cognitive therapy can be a solution. According to this therapy, by increasing the peace of mind and reducing negative thoughts about the disease and futility of treatment, the patients will be more likely to follow their doctor’s advice [18]. The results of several studies showed that this therapy has a positive effect on adherence to doctor’s advice among diabetics and other patients [19–21].

Age of the patients was mentioned as a physical barrier. According to Kriska et al., more physically active people are less likely to develop diabetes, and physical activity decreases by age [22]. Reduced physical activity and deficiency in self-care caused by aging can in turn affect the health level of people [23]. It seems that increasing knowledge and awareness and providing self-care education to patients will help them better comply with treatment, and have a better control over the disease. Identifying and evaluating the needs of people in different age groups and providing appropriate counseling services to patients are two effective solutions in this regard.

More than half of the interviewees stated that they could not act on a given advice because of financial problems. Abdoli et al.’s study showed that participants who were aware of self-care behaviors were overwhelmed by the cost and pressure of getting hold of the medications, controlling blood sugar, and dealing with complications of self-care [24]. In a cross-sectional study, Daly et al. concluded that cost is one of the most common barriers to self-care behaviors [25]. Consistent with our findings, a study conducted in Egypt reported that many of diabetic patients could not buy the monitoring device for regular and prompt detection of fluctuations in their blood glucose levels due to financial matters [26]. The cost of the blood glucose monitoring devices especially in developing countries such as Egypt and Iran needs to be considered as barrier for medication adherence.

Currently, the high consumption of calories, fat, sugar and salt/sodium is increasing among people, and many do not include enough fruits, vegetables and fibers in their diets. A diverse, balanced and healthy diet will vary according to the individual’s needs, cultural backgrounds and food traditions. However, some healthy, diversified, and safe diets need to be considered in every stage of life [27]. Educating patients and promoting a culture for healthy nutrition can help control their disease. Moreover, for patients who cannot afford the food they need, supplementary food packages can be effective in their adherence to medical advice.

The diabetic patients in their interviews stated that lack of family and community support was a barriers of adherence to medical advice. According to Meredith et al., family and the social context in which people live is one of the most effective factors in facilitating the management of disease in diabetic patients. Social support may directly strengthen the immune system by increasing self-esteem and positive emotions, thereby leading to accelerated disease improvement and reduced vulnerability to the disease [28]. In many studies, type 2 diabetic patients performed more self-care behaviors with more family support [29–31]. Another study by Shawon et al. showed that social support and family support were significantly associated with blood glucose control. Measures for public health education must be taken, and family members should be motivated to provide more support to diabetic patients [12]. In this respect, it is possible to help patients act on medical advice by holding training courses for their relatives.

Diabetic patients had wrong beliefs and attitudes about diabetes and they did not take the risk of disease progression seriously, or they felt that adherence to medical advice would be useless. Hasliza Abu et al. concluded that the evaluation of the patient’s concerns and beliefs about diabetes and insulin are very important in helping doctors to provide patient-centered care. Understanding the perceptions of patients, doctors can encourage their patients to take insulin therapy by dealing with changing misconceptions. In addition, continuous education and emotional support can greatly improve the patient’s misconceptions about diabetes [32].

As far as individual factors were concerned, the patients mentioned their type of job as the reason for lack of mobility and physical activity. Physical activity increases mental health and moderates health risk factors such as obesity, high cholesterol, and high blood pressure [33]. Lewis et al. considered inappropriate working conditions as barriers to adherence to treatment. These include not having enough time to eat at work, having difficulty adapting to treatment due to the type of job, and forgetting to take medication due to high work stress [34]. Results of a study conducted in England showed that employees with diabetes were less successful in managing their disease at workplace. In this research, the need to raise managers’ awareness about the economic benefits of supporting the staff who suffer from diabetes has been emphasized [35].

One of the problems that diabetic patients in our study indicated was the lack of sports facilities and inappropriate distribution of facilities in Ahvaz. The Ministry of Health, Welfare and Sports in the Netherlands developed a lifestyle intervention called “BeweegKuur” (Dutch for Exercise Therapy) which incorporated sport and physical activity into the lifestyle of individual patients by focusing on changes in physical activity behavior and diet behavior to prevent and treat type 2 diabetes. The results showed that the program was successful in controlling the disease of the studied patients [36]. Therefore, it is suggested that a program with intersectional cooperation like the one implemented in the Netherlands should be widely used in Iran. The lack of sports facilities can be almost solved by intersectional coordination, planning and cooperation of the municipality with the provision of facilities and urban green spaces for the people.

According to the diabetic patients in our study, publicizing and notifying are very important in adherence to advice. This was considered as a systematic factor. The results of the study of Xianglong et al. showed that anti-smoking television advertising could affect the knowledge about and attitude towards smoking. In other words, television, as one of the most influential mass media, can directly or indirectly influence health-related behaviors among population [37]. Therefore, it is possible to use television and other mass media (radio, newspapers, etc.) to solve the problem of non-adherence to medical advice. Moreover, using other methods of advertising (speech, distribution of pamphlets, brochures, posters, etc.) can be effective in the community [38]. In this study, some patients stated that the diabetes unit and the specialist treatment team did not have a good performance in providing them with information about the disease, which led to not following medical advice. Patients need to get the necessary advice to manage the disease and be motivated to follow the treatment plan through consultation and interaction with the treatment team. Poor performance of the treatment team in meeting patients’ needs and providing incomplete and contradictory information leads patients to experience uncertainty, confusion, dissatisfaction, and sense of non-adherence [39, 40].

Another systemic problem that diabetic patients noted was the lack of appropriate insurance coverage in using the needed healthcare services. Limited income and lack of insurance coverage are commonly perceived as barriers to the effectiveness of self-management education for diabetic patients [38]. In some countries, several measures have been taken to solve the problem of diabetic patients. For example, in 2009, in most parts of Colombia, regulations were approved for private insurance policies, which included special benefits for diabetics such as diabetic medications, equipment, and supplies. In the United States, a government law was passed making compulsory insurance coverage for diabetic patients in three forms of preventive care, including blood glucose monitoring, foot examination, and eye examination [41]. In the plan for the development of the health system in Iran, some actions were taken to improve the insurance coverage for different patients. However, in this study, diabetic patients often stated the high cost of medications and tests were the reasons for their failure in controlling the disease and adhering to medical advice. Accelerating the implementation of the clauses of the health insurance plan that are related to insurance and extension of the scope of tests and services for diabetic patients can help control the disease.

## Limitations

One of the limitations of this study was the reluctance of some patients to be interviewed. They later accepted to be interviewed by explanations given to them regarding the benefits of this study in consultations with the personnel at the diabetes unit.

## Conclusions

Major problems contributing to T2DM patients’ non-adherence to medical advice are divided into two areas of individual and systemic problems. Improving health culture and providing education through mass media and health centers seem to be effective in coping with individual problems and providing self-care and social support for diabetic patients. Implementing targeted interventions in lifestyle, promoting the culture of healthy life through mass media, and particularly, increasing the financial support for services required by diabetics can ultimately lead to improvements in diabetic patients’ adherence to advice. These measures, of course, require an increase in health education activities and the development of inter-sectional health systems as well as a high commitment of society and organizations to improve public health. Policymakers should prioritize the issues raised above.

## Data Availability

All data generated or analyzed during this study are included in this published article.
